# Pharmacokinetic characteristics and anticancer effects of 5-Fluorouracil loaded nanoparticles

**DOI:** 10.1186/1471-2407-8-103

**Published:** 2008-04-15

**Authors:** Su Li, Anxun Wang, Wenqi Jiang, Zhongzhen Guan

**Affiliations:** 1 Department of Medicine, Tumor Hospital, Sun Yat-sen University, Guangzhou, China; 2 Department of Oral and Maxillofacial Surgery, First Affiliated Hospital, Sun Yat-sen University, Guangzhou, China

## Abstract

**Background:**

It is expected that prolonged circulation of anticancer drugs will increase their anticancer activity while decreasing their toxic side effects. The purpose of this study was to prepare 5-fluorouracil (5-FU) loaded block copolymers, with poly(γ-benzyl-L-glutamate) (PBLG) as the hydrophobic block and poly(ethylene glycol) (PEG) as the hydrophilic block, and then examine the 5-FU release characteristics, pharmacokinetics, and anticancer effects of this novel compound.

**Methods:**

5-FU loaded PEG-PBLG (5-FU/PEG-PBLG) nanoparticles were prepared by dialysis and then scanning electron microscopy (SEM) and transmission electron microscopy (TEM) were used to observe the shape and size of the nanoparticles, and ultraviolet spectrophotometry was used to evaluate the 5-FU in vitro release characteristics. The pharmacokinetic parameters of 5-FU/PEG-PBLG nanoparticles in rabbit plasma were determined by measuring the 5-FUby high-performance liquid chromatography (HPLC). To study in vivo effects, LoVo cells (human colon cancer cell line) or Tca8113 cells (human oral squamous cell carcinoma cell line) were implanted in BALB/c nude mice that were subsequently treated with 5-FU or 5-FU/PEG-PBLG nanospheres.

**Results:**

5-FU/PEG-PBLG nanoparticles had a core-shell spherical structure with a diameter of 200 nm and a shell thickness of 30 nm. The drug loading capacity was 27.1% and the drug encapsulation was 61.5%. Compared with 5-FU, 5-FU/PEG-PBLG nanoparticles had a longer elimination half-life (t_1/2_, 33.3 h vs. 5 min), lower peak concentration (C, 4563.5 μg/L vs. 17047.3 μg/L), and greater distribution volume (V_D_, 0.114 L vs. 0.069 L). Compared with a blank control, LoVo cell xenografts and Tca8113 cell xenografts treated with 5-FU or 5-FU/PEG-PBLG nanoparticles grew slower and had prolonged tumor doubling times. 5-FU/PEG-PBLG nanoparticles showed greater inhibition of tumor growth than 5-FU (p < 0.01). In the PEG-PBLG nanoparticle control group, there was no tumor inhibition (p > 0.05).

**Conclusion:**

In our model system, 5-FU/PEG-PBLG nanoparticles changed the pharmacokinetic behavior of 5-FU, thus increasing its anticancer activity. 5-Fluorouracil loaded nanoparticles have potential as a novel anticancer drug that may have useful clinical applications.

## Background

A large body of cancer research has been devoted to the development of targeted anti-neoplastic drugs that are selectively taken up by tumor tissues. Toward this end, researchers have recently developed anti-cancer drugs that are incorporated into polymeric micelles, surface-modified particles, liposomes, or nanoparticles [1-4]. However, there are problems with this general approach, including limited biodistribution, toxic side effects, rapid clearance by the reticuloendothelial system (RES), and limited distribution in the circulation.

Hydrophilic-hydrophobic diblock copolymers have great potential as vehicles for the delivery of anticancer drugs [5-9]. A hydrophobic block forms the inner core, which acts as a drug reservoir, and a hydrophilic block forms the hydrated outer shell, which impedes uptake by the RES [10,11]. The advantages of these copolymers includes solubilization of hydrophobic drugs, sustained release and selective targeting of drugs, and reduced drug interaction with the RES [10,11]. Nanoparticles prepared from poly(γ-benzyl-L-glutamate) (PBLG) and poly(ethylene glycol) (PEG) are a hydrophilic-hydrophobic diblock copolymer that have all of these characteristics [5-9]. PBLG, the hydrophobic moiety, is biodegradable and acts as a drug incorporation site [12]. PEG, the hydrophilic moiety, is a non-toxic, non-immunogenic hydrophilic polymer that prevents interactions with cells and proteins [13].

5-Fluorouracil (5-FU), a pyrimidine analogue that interferes with thymidylate synthesis, has a broad spectrum of activity against solid tumors. However, 5-FU has limitations that include a short biological half-life due to rapid metabolism, incomplete and non-uniform oral absorption due to metabolism by dihydropyrimidine dehydrogenase [14-17], toxic side effects on bone marrow and the gastrointestinal tract, and non-selective action against healthy cells [18].

In order to prolong the circulation time of 5-FU and increase its efficacy, numerous researchers have attempted to modify its delivery by use of polymer conjugates or by incorporation of 5-FU into particulate carriers [19-23]. The ultimate aim of these strategies is to reduce 5-FU associated side effects and thereby improve its therapeutic index [19-23]. In this study, we used a diafiltration method to prepare 5-FU-loaded PEG-PBLG (5-FU/PEG-PBLG) nanoparticles and evaluate their physical characteristics, in vitro release behavior, and anti-tumor activity.

## Methods

### Preparation of PEG-PBLG

PEG-PBLG block copolymers (MW, 1.12 × 10^4^) were prepared by polymerization of γ-benzyl-L-glutamate N-carboxyanhydride (γ-BLG NCA) initiated with mono amine-terminated PEG in a methylene dichloride solution, as described previously [24]. Briefly, we prepared the monoamino-terminated poly(ethylene glycol) (MeO-PEG-NH_2_) by the use of toluene sulfonate esterification with MeO-PEG-OH. The production rate of this process was 51.9 % and the transformation rate was 68.2%. The γ-benzyl-L-glutamate was obtained by reaction of glutamic acid with benzyl alcohol at 120°C for 5 h under 60% sulfuric acid (activator), and then reacted with triphosgene to obtain the monomer of γ-benzyl-L-glutamate N-carboxyl anhydride (BLG-NCA). The process production rate was 53.2%. The amphiphilic block copolymer was the prepared by anionic polymerization of BLG-NCA initiated by MeO-PEG-NH_2 _with a 50/1 molar ratio of monomer/initiator. The resulting molecular weight was 1.12 × 10^4^. IR and 1H-NMR demonstrated that MeO-PEG-NH_2 _was polymerized with BLG-NCA to form PEG-PBLG.

### Drug and Chemicals

5-FU was purchased from Sigma (USA). Other chemicals were of laboratory grade purity.

### Cell Culture

Human colon cancer cells (LoVo cell line) and human oral squamous carcinoma cells (Tca8113 cell line) were grown in RPMI 1640 medium (GIBCO) with 10% fetal calf serum (GIBCO), 100 units/ml penicillin G, and 100 μg/ml streptomycin at 37°C in 5% CO_2_.

### Animals

New Zealand rabbits (2–3 kg) and BALB/c nude mice (6–8 weeks old, 20–30 g) were purchased from the animal center at Sun Yat-sen University (Guangzhou, China). All animal experiments were performed with permission of the Animal Ethical Commission of Sun Yat-sen University.

### Preparation and identification of 5-FU/PEG-PBLG nanoparticles

5-FU/PEG-PBLG nanoparticles were prepared by a diafiltration method. Briefly, we dissolved PEG-PBLG diblock copolymers and 5-FU (1:1 w/w) in dimethylformamide (DMF) and dialyzed the solution (with a molecular weight cut-off of 3500 g/mol; Spectrum Medical Industries, Inc., Houston, TX) in double-distilled water for 24 h. The solution inside the dialysis bags was collected and then centrifuged (2000 rpm/min; 10 min). The supernatant (nanoparticles) was filtered with a 0.45 μm filter. The samples were then freeze-dried for subsequent use. A 640 UV spectrophotometer (Bechman) was used to identify the 5-FU/PEG-PBLG nanoparticles by scanning from 200 nm to 400 nm.

### Morphology of PEG-PBLG nanoparticles

A scanning electron microscope (HITACH-600, Japan) and a transmission electron microscope (PHILIPS, Holland) were used to examine particle morphology. For SEM, PEG-PBLG samples were filtered with a 0.45 μm sieve and dropped onto a slide. The prepared samples were dried at room temperature for several days and then gilded. The final concentration of the gilded samples was 0.2 mg/ml. For TEM, one drop of the PEG-PBLG sample was added to a copper supported mesh membrane and the excess solution removed with filter paper. Then, 1% phosphotungstic acid was added to the mesh membrane. Excess solution was removed after 1 minute and the sample dried at room temperature. The concentration of prepared sample was 0.2 mg/ml.

### Loading capacity, drug encapsulation, and in vitro release

5-FU/PEG-PBLG nanoparticles were placed into dialysis bags and the bags were introduced into a DMF solution. After stirred at 37°C for 3 h dialysed sample was determined for drug concentration by measuring absorbance at 269 nm. The drug loading capacity and drug encapsulation were calculated by the following formulas:

Drug loading capacity = M_5-FU_/M_5-FU/PEG-PBLG_

Drug encapsulation = M_5-FU_/M_drug devoted_

where M_5-FU _was the drug content detected in solution [M_5-FU _= D_5-FU _× V, D_5-FU _= (A_sample_/A_standard_) × D_standard_, D: concentration, V: volume]; M_5-FU/PEG-PBLG _was quantity of 5-FU/PEG-PBLG nanoparticles detected in solution; and M_drug devoted _was the initial quantity of 5-FU.

For in vitro release studies, 5-FU/PEG-PBLG nanoparticles were placed into dialysis bags and the bags were introduced into PBS at pH 6.86 or pH 9.18. The medium was stirred at 94 ± 4 beats/min at 37°C. The medium was replaced with fresh PBS at variable periods of time up to 96 h. We determined the concentration of 5-FU that was released into the PBS by measuring the absorbance at 269 nm.

### Pharmacokinetic studies of 5-FU/PEG-PBLG nanoparticles in rabbit plasma

A single dose of 5-FU or 5-FU/PEG-PBLG nanoparticles (30 mg/kg) was administered to rabbits. Blood samples were collected from rabbit veins at designated times after intravenous administration. 5-FU was extracted from plasma by mixing rabbit plasma with ethyl acetate and isopropyl alcohol (85/15, v/v). The samples were then dried with N_2 _at 37°C and the dehydrated samples were dissolved in 400 μl of mobile phase dilutent for subsequent HPLC.

The concentration of released 5-FU was measured using reversed-phase HPLC (HP1100 Liquid Chromatogragh, Agilent). A Hypersil C18 (5 μm, ID 4.6 mm × 300 mm) analytical column was used with a mobile phase of 0.01 mol/L phosphate buffer (pH 3.0) and an elution rate of 1.0 ml/min at room temperature. Absorbance at 269 nm was monitored and pharmacokinetic parameters were determined from the absorbance-time curves. This method provided complete separation with a corresponding retention time of 7.0 minutes for 5-FU. The standard calibration curve of 5-FU absorbance with concentration was y = 3.47x + 0.24 (γ > 0.9998). The lower limit of determination was 5 μg/L.

### In vivo tumor inhibition effect of 5-FU loaded nanoparticles

LoVo cells were subcutaneously implanted in the right flank of BALB/c nude mice. Mice were assigned to one of 4 groups (n = 8) after xenografts were about 5 mm in diameter: 1) control group (PBS), 2) PEG-PBLG group, 3) 5-FU group, 4) 5-FU/PEG-PBLG nanoparticle (3 mg/kg) group. For groups 3 and 4, intraperitoneal injections were administered daily for 7 days. For groups 1 and 2, intraperitoneal injections of the same volume of PBS or PEG-PBLG were administered on the same schedule. The mice were sacrificed on day 21 and tumor size was measured (length and width) with a caliperevery 3 days. Tumor parameters were calculated on day 21 by the following formulas: Tumor volume = (1/2 × length × width^2^); Tumor doubling time = (ln2/K where K = growth rate); Inhibition rate at day 21 = (1- volume change of experimental group/volume change of control group) × 100%.

For the mice with implanted Tca8113 cells, 5-FU or 5-FU/PEG-PBLG (3 mg/kg) was injected intraperitoneally every 2 days for 16 days. Mice were sacrificed on day 34.

### Statistical analysis

All experiments were performed in triplicate and data are presented as mean ± SD. The tumor growth inhibitory effect of drugs was analyzed using one-way analysis of variance. P < 0.05 was considered as statistically significant.

## Results and Discussion

### Characteristics of 5-FU/PEG-PBLG nanoparticles

**Figure 1:**
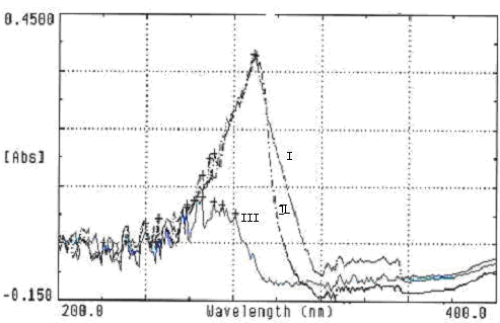
**UV spectra of 5-FU/PEG-PBLG nanoparticles**. I: 5-FU; II: 5-FU + PEG-PBLG nanoparticles; III: 5-FU/PEG-PBLG nanoparticles. I and II had high absorbance at 269 nm while the absorbance of III was much lower at 269 nm.

Drug-loaded nanoparticles have a diameter ranging from 10 nm to 500 nm and incorporate a drug by conjugation, physical entrapment, absorption, or by other mechanisms. Nanoparticles can enhance the solubilization of a hydrophobic drug, protect drug activity, increase drug stability, improve the drug's therapeutic index, and decrease adverse side effects [2-9]. Drug-loaded nanoparticles are widely used for anticancer drugs because of their enhanced targeting properties [2-9,25]. Previously, researchers have used copolymers of PEG and PBLG, a biocompatible and biodegradable macromolecular material, as the nanoparticle carrier for a drug [5-9]. In this study, we prepared 5-FU/PBLG-PEG nanoparticles by a diafiltration method. Analysis by UV spectrophotometry (Figure 1) showed that a solution of 5-FU, or a simple mixture of 5-FU and PBLG-PEG nanoparticles, had high absorbance at 269 nm. However, the absorbance of 5-FU/PEG-PBLG nanoparticles at 269 nm had greatly decreased. This indicates that 5-FU can be loaded into PEG-PBLG nanoparticles by diafiltration. Based on the decrease in absorbance at 269 nm, we estimate the drug loading capacity as 27.1% and the drug encapsulation as 61.5%.

Experiments to determine drug release and pharmacokinetics were performed to demonstrate the reliability of the aforementioned observation. It's also of note that the drug loading capacity in our study was higher than the 10% achieved by Nagaich et al. [19] who prepared 5-FU loaded PEG-polysaccharide nanoparticles. Whether the difference is due to the PBLG component, which has a steel-like structure and forms a hydrophobic core, remains to be determined [26-28].

**Figure 2:**
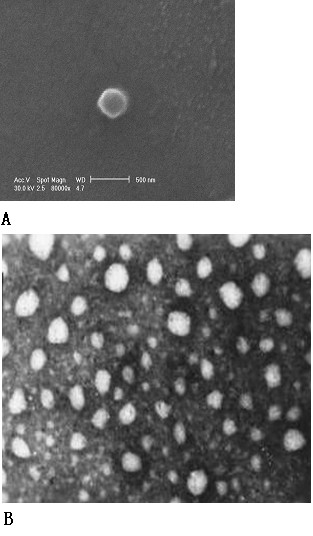
**The core-shell structure of 5-FU/PEG-PBLG nanoparticles. (A) Morphology under SEM (× 80000)**. SEM showed 5-FU/PEG-PBLG nanoparticles have a core-shell structure, a spherical or elliptical shape, and a smooth surface. The hydrophobic central core is a non-gilt grizzly area, about 200 nm in diameter and the hydrophilic shell is a gilt white area, about 30 nm in thickness. **(B) Morphology under TEM (× 50000)**. TEM showed that nanomicelles were round or oval particles of uniform size with fuzzy edges.

Like many other polymeric nanoparticles [5-9], the morphology of our 5-FU/PEG-PBLG nanoparticles is spherical or elliptical, with a core-shell structure, and a smooth surface (Figure 2). The hydrophobic PBLG central core is a non-gilt grizzly area, about 200 nm in diameter, and the PEG hydrophilic shell is a gilt white area, approximately 30 nm in thickness (Figure 2A). A representative TEM scan of the 5-FU/PEG-PBLG nanoparticle is shown in Figure 2B. Previous research has shown that nanoparticles are not easily phagocytized when the thickness of the PEG layer is 10 nm for every 100 nm thickness of the micelles. This indicates that the RES should not take up the 5-FU/PEG-PBLG nanoparticles examined in this study.

### In vitro release of 5-FU loaded nanoparticles

**Figure 3:**
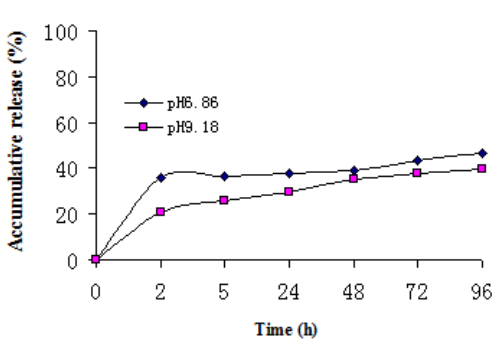
In vitro release of 5-FU from PEG-PBLG nanoparticles at pH 6.86 and pH 9.18.

Nanoparticle release occurs by 2 methods: "burst release" and "sustained release" [29-32]. Burst release is the rapid release of a drug from the surface of nanoparticles or diffusion from the polymer matrix. This allows the drug to rapidly reach an effective concentration in the circulation. Sustained release is the slow release of a drug that is entrapped within nanoparticles during nanoparticle biodegradation. This allows the drug to stay at an effective concentration in the circulation over time. Figure 3 shows the in vitro 5-FU release profiles from PEG-PBLG nanoparticles at pH 6.86 and pH 9.18. This brackets the normal pH of human blood (pH ~7.4). The 5-FU release profiles are composed of burst release and sustained release [33]. The burst release, which resulted in the release of ~30% of the 5-FU, occurred from 0 to 2 h. The sustained release occurred from 2 to 96 h and resulted in the release of ~50% of the 5-FU.

### Pharmacokinetics characteristic of 5-FU/PEG-PBLG nanoparticles in vivo

Clinically, 5-FU can be administered by bolus injection, which primarily inhibits RNA synthesis, or by continuous infusion, which primarily inhibits DNA synthesis. Clinical response would be expected to be enhanced if both methods could be combined [16,17]. The half-life of 5-FU in vivo is only 5 to 10 min; thus, many studies have attempted to develop 5-FU preparations with a prolonged lifetime [21,32].

**Figure 4:**
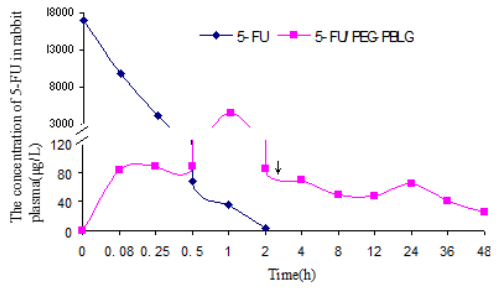
**Mean plasma concentration of 5-FU following a single i.v. administration of 5-FU or 5-FU/PEG-PBLG nanoparticles at 30 mg/kg**. The arrow depicts sustained release.

To study the in vivo characteristics of the 5-FU/PEG-PBLG nanoparticles, we administered 5-FU or 5-FU/PEG-PBLG nanoparticles to rabbits at a single dose of 30 mg/kg. The absolute recovery and relative recovery of 5-FU were 73.5 to 82.6% and 98.6 to 100.8%, respectively. The intra- and inter-day RSD (relative standard deviation) was less than 10%, justifying this method for study of 5-FU pharmacokinetics in rabbits. As Figure 4 shows, 5-FU alone followed a one-compartment model, but 5-FU/PEG-PBLG nanoparticles followed a multi-compartment model with a burst release followed by a sustained release. Compared with 5-FU, 5-FU/PEG-PBLG nanoparticles have a greater elimination half-life (t_1/2_), lower peak concentration (C_max_), greater distribution volume (T_max_), and slightly lower area under the curve (AUC) (Table 1). This indicates that when 5-FU is loaded into nanoparticles, the 5-FU has sustained-release, prolonged half-life, and increased tissue appetency. It is noteworthy that the pharmacokinetic characteristics of our 5-FU loaded PEG/PBLG nanoparticles are similar to the 5-FU loaded PEG-polysaccharide nanoparticles of Nagaich et al. [19].

**Table 1:** In vivo pharmacokinetic parameters of 5-FU and 5-FU/PEG-PBLG nanoparticles

	**t_1/2_(h)**	**C_max _(μg/L)**	**T_max_(h)**	**V_d_(L)**	**AUC(μg · h/L)**
**5-FU**	0.088	17047.3	0	0.069	6263.7
**5-FU/PEG-PBLG**	33.3	4563.5	1.25	0.114	5794.7

t_1/2_, elimination half-life; C_max_, peak concentration; T_max_, peak time; AUC, area under the concentration-time curve; V_d_, distribution volume.

### Anticancer effect of 5-FU loaded nanoparticles in vivo

**Figure 5:**
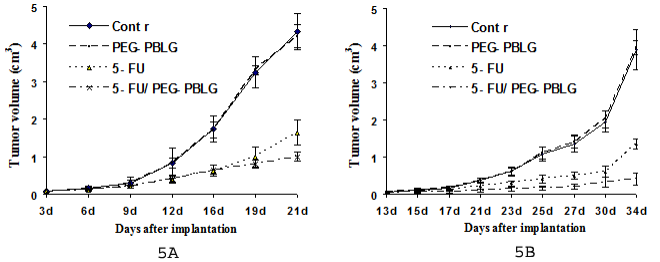
Tumor growth of LoVo cell xenografts (A) and Tca8113 cell xenografts (B) after treatment with 5-FU or 5-FU/PEG-PBLG nanoparticles.

5-FU is clinically effective against human colorectal cancer and oral squamous cell carcinoma [14]. Figures 5A and 5B show that LoVo cell xenografts (colorectal cancer) and Tca8113 cell xenografts (oral squamous carcinoma) grew rapidly in blank and in the PEG-PBLG control groups. However, 5-FU and 5-FU/PEG-PBLG nanoparticles significantly inhibited tumor growth. Tumor doubling times and inhibition rates are presented in Table 2. There were no differences between the 2 control groups (p > 0.05), but there were significant differences between the 2 treated groups (p < 0.01). We observed no significant toxicity in any group. The more effective anticancer effect of 5-FU/PEG-PBLG nanoparticles (Figure 5, Table 2) may be due to: 1) the sustained-release, prolonged half-life, and increased apparent volume of distribution of 5-FU/PEG-PBLG nanoparticles and/or 2) the neovascularization and higher permeability of blood vessels present in tumor cells, making it easier for nanoparticles to enter tumor cells and thereby increase the anticancer effect of 5-FU.

**Table 2:** The anticancer effect of 5-FU/PEG-PBLG nanoparticles

	**LoVo cell xenografts**			**Tca8113 cell xenografts**		
	**TDT (d)**	**IR (%)**	**TV(cm^3^)**	**TDT (d)**	**IR (%)**	**TV(cm^3^)**

**Blank control**	3.0	0	4.336 ± 0.485	3.5	0	3.888 ± 0.547
**PEG-PBLG**	2.9	0	4.206 ± 0.308*	3.6	0	3.944 ± 0.179*
**5-FU**	4.08	62.2%	1.637 ± 0.330^#^	4.6	65.4%	1.346 ± 0.142^#^
**5-FU/PEG-PBLG**	4.50	77.1%	0.993 ± 0.122^#§^	5.3	89.6%	0.405 ± 0.174^#§^

TDT, tumor doubling time; IR, inhibition rate; TV, tumor volume at day 21 for LoVo cell xenografts and at day 34 for Tca 8113 cell xenografts.

*compared with blank control, p > 0.05.

#compared with blank control, p < 0.01,

§compared with 5-FU, p < 0.01.

## Conclusion

In this study, we prepared 5-FU loaded PEG-PBLG nanoparticles (5-FU/PEG-PBLG) which exhibited favorable pharmacokinetic characteristics, including sustained drug release, prolonged drug half-life, and increased tissue appetency. In vivo, 5-FU/PEG-PBLG nanoparticles had good anti-tumor activity against colon cancer xenografts and oral squamous cell carcinoma xenografts. Taken together, our results indicate that a PEG-PBLG nanoparticle delivery system for 5-FU may be able to effectively reduce adverse side effects of 5-FU therapy and improve the therapeutic index of 5-FU.

## List of abbreviations

5-FU, 5-fluorouracil; PBLG, poly(γ-benzyl L-glutamate); PEG, poly(ethylene glycol); SEM, scanning electron microscopy; HPLC, high-performance liquid chromatography; RES, reticuloendothelial system, DMF, N,N-dimethylformamide; t_1/2_, elimination half-life; C_max_, peak concentration; T_max_, peak time; AUC, area under the concentration-time curve; V_D_, distribution volume; TDT, tumor doubling time; IR, inhibition rate; TV, tumor volume at day 21 for LoVo cell xenografts or day 34 for Tca 8113 cell xenografts.

## Competing interests

The author(s) declare that they have no competing interests

## Authors' contributions

SL and AW were responsible for experimental design and completion of all laboratory work. WJ and ZG participated in the design and coordination of the work. SL and AW wrote the manuscript. All authors have read and approved the final manuscript.

## Pre-publication history

The pre-publication history for this paper can be accessed here:


